# Assessment of oxidative stress-induced cellular damage in preeclamptic females

**DOI:** 10.3389/fmed.2026.1892937

**Published:** 2026-08-19

**Authors:** Simmi Kharb, Gurpreet Singh Gill, Nirmala Bhandari, Anjali Gupta

**Affiliations:** 1 Department of Biochemistry, Pt. B.D. Sharma PGIMS, Rohtak, Haryana, India; 2 Department of Obstetrics & Gynecology, Pt. B.D. Sharma PGIMS, Rohtak, Haryana, India

**Keywords:** biomarker, LDH, oxidative stress, preeclampsia, pregnant, uric acid

## Abstract

**Introduction:**

Preeclampsia is a hypertensive disorder of pregnancy characterized by the development of hypertension and proteinuria after 20 weeks of gestation. Oxidative stress, arising from an imbalance between reactive oxygen species (ROS) production and antioxidant defenses, has been implicated in the pathogenesis of preeclampsia.

**Methods:**

This study aimed to assess oxidative stress-induced cellular damage in preeclamptic females through the evaluation of oxidative stress biomarkers and their association with disease severity. Blood samples were collected from preeclamptic females and normotensive pregnant controls matched for gestational age. Routine biochemical investigations, including serum uric acid and lactate dehydrogenase (LDH) levels, were measured.

**Results:**

The results revealed significantly elevated levels of oxidative stress biomarkers in preeclamptic females compared to normotensive controls. Serum LDH levels were significantly increased in preeclampsia, and with the severity of the disease, the levels were higher. Serum LDH levels showed a significant correlation with serum uric acid levels. Furthermore, a positive correlation was observed between oxidative stress parameters and disease severity, as indicated by blood pressure levels, transaminases, and proteinuria.

**Discussion:**

These findings underscore the importance of oxidative stress-induced cellular damage in the pathophysiology of preeclampsia and highlight the potential utility of oxidative stress biomarkers as diagnostic and prognostic indicators in this hypertensive disorder of pregnancy. Further research is warranted to elucidate the underlying mechanisms linking oxidative stress to preeclampsia and explore novel therapeutic strategies targeting oxidative stress pathways in the management.

## 1 Introduction

Among the hypertensive disorders of pregnancy, preeclampsia (PE) is a particularly serious form that can profoundly impact both pregnant women and fetuses. This disease encompasses 2% to 8% of pregnancy-related complications, more than 50,000 maternal deaths, and over 500,000 fetal deaths worldwide. The parameters for initial identification of hypertension in the context of pregnancy-induced hypertension include a systolic blood pressure (SBP) of 140 mm Hg or more or diastolic blood pressure (DBP) of 90 mm Hg or more on 2 occasions at least 4 h apart (as endorsed by the American College of Obstetrics and Gynecology).

The fundamental pathological and physiological alterations associated with PE involve systemic small vessel spasms and endothelial injury, stemming from a complex interplay of various factors, mechanisms, and pathways. Despite extensive research, the precise etiology and pathogenesis of PE remain incompletely understood to this day. Oxidative stress (OS) has emerged as a central contributor among the mechanisms most strongly linked to hypertensive disorders of pregnancy. Excessive production of reactive oxygen species (ROS) contributes to DNA damage, apoptosis, endothelial dysfunction, increased release of pro-inflammatory cytokines, and impaired anti-inflammatory responses (1).

A high level of oxidative stress can cause lipids and proteins to oxidize, which results in alterations to their structure and functions (1). Numerous disorders, such as atherosclerosis, inflammatory diseases, some malignancies, and the aging process, have been linked to oxidative stress. Today, it is believed that oxidative stress plays a major role in the development of all inflammatory illnesses, ischemic diseases, emphysema, hypertension, preeclampsia, neurological disorders, and many other conditions (2). The use of the total radical-trapping antioxidant parameter (TRAP) has recently been proposed to explore the antioxidant property of a plasma sample. TRAP may be either directly measured by a fluorescence-based method (TRAPm) (3) or calculated (TRAPc) by a mathematical formula, from serum levels of four natural antioxidants: protein-bound SH (thiol) groups, uric acid, vitamin E, and vitamin C (4). TRAPc, easier to measure than TRAPm, might be adequately reliable for routine assessment of oxidative stress (5).

Uric acid has a dual action, both as an antioxidant and pro-oxidant (6). Uric acid-induced ROS production seems paradoxical since uric acid has generally been considered to be one of the important antioxidants that protect from oxidative stress (7). Uric acid acts as an antioxidant only in the hydrophilic environment. The differential role of hyperuricemia suggests the presence of a molecular switch that controls the role of uric acid as an antioxidant vs. a pro-oxidant (8). Uric can scavenge reactive radicals released into the blood by autoxidation of hemoglobin or peroxide production by macrophages (9). However, even in the plasma urate can prevent lipid peroxidation (10). Regulator enzyme of uric acid production in mammals is synthesized as a dehydrogenase (XDH), which uses NAD^+^ as the electron acceptor, but it can be converted into oxidases (XO) which use molecular oxygen as the electron acceptor (11). Conversion into xanthine oxidase (XO) may occur through sulfhydryl group oxidation, which can be reversed with thiol reagents, e.g., dithiothreitol (DTT) (12). Reversible XDH to XO conversion has been attained experimentally by glutathione depletion (13), ischemia (14), ischemia-reperfusion, and oxidizing agents (15). Irreversible conversion occurs by Proteolytic cleavage of 15–20 kDa fragments of XDH subunits during tissue ischemia (16). The proteolytic conversion has been hypothesized to be modulated by intracellular calcium levels (17). Calcium-dependent proteolytic XDH to XO conversion is sensitive to calmodulin inhibitors (18), but the enzyme responsible for the conversion is unknown.

Hyperuricemia has been more prevalent, and its burden is rendered heavier by its relationship with numerous comorbidities, such as cardiovascular disease, hypertension, etc. (19). A recent meta-analysis reveals that a 1 mg/dL increase in serum uric acid level is associated with a significant increase in the incidence of hypertension (relative risk 1.13, 95% confidence interval) (20).

Preeclampsia (PE)/gestational hypertension (GHTN) is one of the most prevalent and feared consequences with an unclear origin, although its main symptoms are thought to be ischemia due to faulty placentation and endothelial dysfunction (21). Endothelial dysfunction caused by increased ROS generation plays a significant role in this phenomenon in several different ways, mainly the interaction of ROS with NO, which is the best- characterized (and possibly most significant) modulator of endothelial function (22).

Quantification of serum LDH level has a clinical interest as it leaks out of the cytoplasm due to loss of integrity of the plasma membrane due to lipid peroxidation; thus, there is an increased level of serum LDH in oxidative stress (23).

According to recent investigations, even asymptomatic young people with primary hyperuricemia exhibited much more oxidative stress than healthy people (24). Therefore, the goal of the present study was to correlate the levels of uric acid in preeclamptic females with serum LDH levels, which rise as a result of oxidative stress-induced cellular damage.

## 2 Materials and methods

### 2.1 Population and design of the study

The department of Biochemistry at Pt. B.D. Sharma PGIMS, Rohtak, conducted this case-control study on 100 pregnant women (28 weeks) with preeclampsia who visited Obstetrics OPD (Haryana, India). Preeclampsia was identified by proteinuria (24 h urine collection >300 mg protein or single voided urine protein/creatinine ratio ≥0.3; Dipstick reading of 2+) and elevated systolic and diastolic blood pressure as per guidelines of the American College of Obstetrics and Gynecology (25). After receiving consent, 100 normotensive pregnant women of the same age range and socioeconomic background were enrolled in the control group.

### 2.2 Technique for sampling and biochemical analysis

A detailed history and clinical examination were recorded for each individual, and 5 mL of venous blood was drawn from each individual, preferably in the morning after overnight fasting under aseptic conditions in a plain vial for biochemical analysis. Centrifugation was done to separate the serum before using it for routine biochemical investigations, including serum uric acid levels, LDH enzyme activity, alanine transaminase enzyme activity, and aspartate transaminase enzyme activity as per standard methods (enzymatically) of a fully automatic Randox analyzer (26).

### 2.3 Statistical analysis

The data were analyzed with Student's independent *t*-test and Pearson Correlation coefficient on Microsoft Excel, and *P*<*0.05* was accepted as statistically significant.

## 3 Observations

The average age of the study group and control was 24.38 ± 3.68 years (range from 18–35 years) and 25 ± 2.99 years (range from 19–34 years) respectively and they were comparable. Based on systolic and diastolic blood pressure, the 100 study participants were further divided into two categories: those with mild preeclampsia (51 females) and those with severe preeclampsia (49 females). When compared to the Control group, there was a strong correlation between both categories of the study group's blood pressure and preeclampsia (*p* = *0.0001*) (Table 1).

**Table 1:** Distribution of cases & control group.

Parameters	Cases		Control	*p*-value	Significance level
	Mild preeclampsia	Severe preeclampsia			
Systolic B.P.	147.17 ± 7.76	164.63 ± 8.18	116.56 ± 7.95	* ≤ 0.0001*	Highly Significant
Diastolic B.P.	97.78 ± 4.67	109.48 ± 6.50	77.42 ± 7.54	* ≤ 0.0001*	Highly Significant

Showing Mean ± SD of systolic and diastolic blood pressure of cases & control and correlation between them.

Females with mild preeclampsia had lower levels of serum uric acid as compared to severe preeclampsia (5.75 ± 1.95 mg/ dL vs. 7.91 ± 1.99 mg/dL, respectively), and as opposed to 4.52 ± 1.64 mg/dL in the control group. The correlation of serum uric acid levels between mild preeclampsia and control was significant (*p* = *0.0001*) (95% CI of −1.8321 to −0.6279). The correlation between severe preeclampsia and control has *P* = *0.0001* (95 percent confidence interval [CI] of −3.9902 to −2.7898) and is very highly significant.

The mean LDH level in mild and severe preeclampsia was 531.73 ± 168.33 and 733.05 ± 236.95 IU/L, respectively. The mean LDH level of the study group's two categories was 630.38 ± 227.52 IU/L, which was higher than the control group's mean LDH level of 224 ± 116.61 IU/L. With a *P*-value of 0.0001, the association between the average LDH levels in the study group and the control group is very highly significant (95 percent CI of −453.8098 to −352.0902).

Serum LDH levels were compared with serum uric acid levels by using Pearson's correlation in 100 preeclamptic patients (Table 2). The R-value for Pearson's correlation was 0.4066. The result was significant since the coefficient of determination (R^2^) had a value of 0.1653, and the *p*-value was 0.00027 at a significance level of 0.05.

**Table 2:** Pearson's correlation between serum uric acid and LDH.

Parameters	Uric acid (mg/dl)	LDH (IU/L)	R-value	R^2^ Value	*p*-value
Preeclampsia	6.816 ± 2.24	630.38 ± 227.52	0.406639	0.1653	0.00027

Showing the Coefficient of Pearson's correlation (R), Coefficient of determination (R^2^), and *P*-value of a positive correlation between serum uric acid & LDH level in pregnant females with preeclampsia.

Serum AST and ALT levels were also measured in patients (Table 3) along with uric acid and LDH. These parameters were compared with age and systolic blood pressure, and also with each other, in mild and severe preeclamptic females. In mild preeclampsia, AST had a high positive correlation (R = 0.7938), (R = 0.5699) and low positive correlation (R = 1897) with ALT, Uric acid, and LDH, respectively. ALT also had a high positive correlation (R = 0.5167) and a low positive correlation (R = 2497) with uric acid and LDH, respectively. Uric acid and systolic blood pressure (SBP) also had a low positive correlation (R = 0.2520 and R = 0.2010) with LDH in mild preeclampsia. A low negative correlation (−0.2279) was found between age and LDH level (Table 4).

**Table 3:** Mean ± SD of level various biochemical parameters.

Parameters	Mild preeclampsia	Severe preeclampsia	*p*-value	Level of significance
AST (IU/L)	55.86 ± 61.40	81.11 ± 55.68	* ≤ 0.01*	Significant
ALT (IU/L)	53.37 ± 53.94	84.80 ± 66.87	* ≤ 0.01*	Significant
UA (mg/dl)	5.75 ± 1.95	7.91 ± 1.99	* ≤ 0.01*	Significant
LDH (IU/L)	531 ± 168.33	726.93 ± 244.53	* ≤ 0.001*	Highly Significant

Showing level of serum AST, ALT, uric acid (UA), and LDH in mild and severe preeclampsia and their level of statistical significance.

**Table 4:** Pearson's correlation between various variables in mild preeclampsia.

Parameters	Age	AST	ALT	UA	LDH	SBP
Age	-	−0.0166	−0.0415	0.0631	−0.2279	−0.0791
AST	−0.0166	-	**0.7938**	**0.5699**	**0.1897**	−0.1269
ALT	−0.0415	**0.7938**	-	**0.5167**	**0.2497**	−0.0287
UA	0.0631	**0.5699**	**0.5167**	-	**0.2520**	−0.1532
LDH	−0.2279	**0.1897**	**0.2497**	**0.2520**	-	**0.2010**
SBP	−0.0791	−0.1269	−0.0287	−0.1532	**0.2010**	-

Showing R-value of Pearson's correlation of Age, AST, ALT, uric acid (UA), LDH, and SBP (Systolic Blood Pressure) with each other in 48 patients with mild preeclampsia. Bold values indicate statistically strong correlation.

In females with severe preeclampsia, AST had a high positive correlation (R = 0.9107), (R = 0.54240) and low positive correlation (R = 0.2395) with ALT, LDH, and uric acid, respectively. ALT had a high positive correlation with LDH (R = 0.5312) and a moderate positive correlation (R = 0.3657) with uric acid. Uric acid also had a low positive correlation (0.2441) with LDH. A low positive correlation was found between systolic blood pressure, LDH, and uric acid. In severe preeclamptic females, age had a low negative correlation with ALT, uric acid, and LDH (Table 5).

**Table 5:** Pearson's correlation between various variables in severe preeclampsia.

Parameters	Age	AST	ALT	UA	LDH	SBP
Age	-	−0.0964	−0.1545	−0.1517	−0.2521	0.0719
AST	−0.0964	-	**0.9107**	**0.2395**	**0.5424**	0.0344
ALT	−0.1545	**0.9107**	-	**0.3657**	**0.5312**	0.0515
UA	−0.1517	**0.2395**	**0.3657**	-	**0.2441**	**0.1577**
LDH	−0.2521	**0.5424**	**0.5312**	**0.2441**	-	**0.1855**
SBP	0.0719	0.0344	0.0515	**0.1577**	**0.1855**	-

Showing R-value of Pearson's correlation of Age, AST, ALT, uric acid (UA), LDH, and SBP (Systolic Blood Pressure) with each other in 52 patients with severe preeclampsia. Bold values indicate statistically strong correlation.

The patients and controls were divided into three groups based on the period of gestation (weeks) at the time of enrollment. The results were insignificant (p ≥ 0.05) when gestation periods were correlated with control (Table 6).

**Table 6:** Period of gestation at the time of enrollment.

Period of gestation (Weeks)	Frequency in study group	Frequency in control group	Statistical significance
28–32	25	9	*p≥0.05* insignificant
32–36	36	23	
≥36	39	68	

Showing period of gestation of 100 patients and 100 controls at the time of enrollment and statistical significance of the period of gestation in case and control.

## 4 Discussion

A case-control study involving 100 pregnant women with preeclampsia was done. The mean age of these females was 24.38 ± 3.68 years (ranging from 18–35 years). A group of 100 normotensive pregnant females was taken as control with an average age of 25 ± 2.99 years (range from 19–34 years). In a study conducted by Bhave et al. (27), it was also observed that the mean age for mild preeclampsia and severe preeclampsia was remarkably similar to the mean age of the present study group. The study group of 100 patients was divided into two categories, i.e., mild preeclampsia and severe preeclampsia, based on systolic and diastolic blood pressure. Since increased blood pressure is one of the chief complications of preeclampsia thus the mean comparison of blood pressure of control and cases was done by *t*-test which was very highly significant (*p* ≤ *0.0001*).

**Figure 1:**
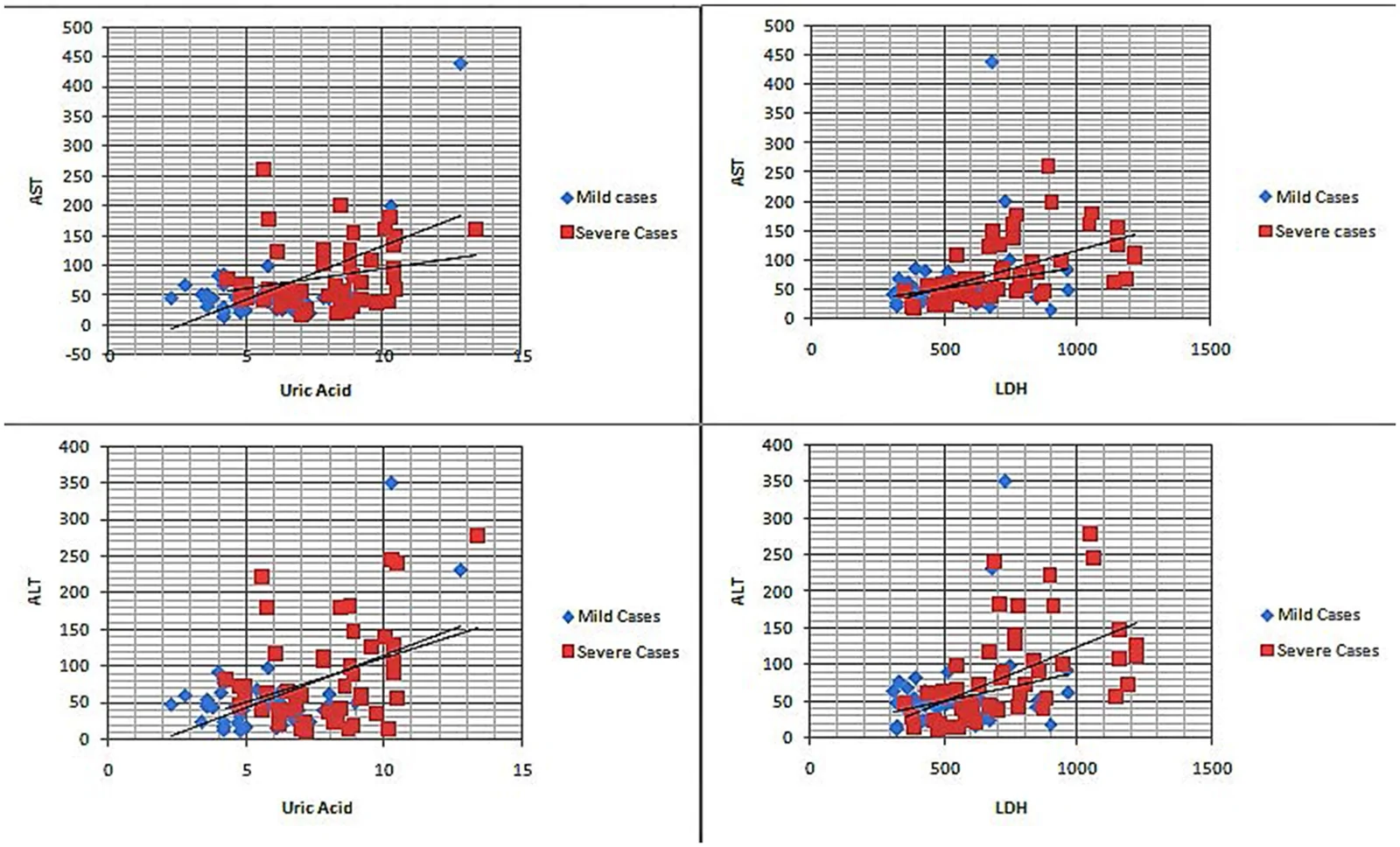
Scattered graph of Pearson's correlation. Showing Scattered graphs of correlation among ALT, AST, uric acid, and LDH in mild and severe cases of preeclampsia.

As a byproduct of the metabolism of purines, uric acid is produced by xanthine oxidase, which also produces the free radical, which is linked to oxidative stress. A typical finding in preeclamptic pregnancies is hyperuricemia. In preeclamptic women, an increase in uric acid frequently occurs before hypertension and proteinuria (27). In the present study, the mean uric acid level in mild and severe preeclampsia was increased compared to the statistically significant control group. In a study conducted by Ryu et al. (28), similar results were found. This same study also found that serum uric acid levels were positively correlated with systolic blood pressure in individuals with preeclampsia (R = 0.321), which was quite similar to the finding in our study (R = 0.361) (Figure 1). Another study by Bellomo et al. (29) concluded that blood uric acid levels ≥309 μmol correctly predicts the later onset of preeclampsia in pregnant women. In this study, a similar pattern was followed which leads to mild preeclampsia in early pregnancy and later onset of severe eclampsia.

Although the exact cause of hyperuricemia in preeclampsia has not yet been identified, current research suggests that uric acid may serve as an early indicator of the condition. Hyperuricemia is caused by increased placental uric acid production as a result of placental ischemia and increased trophoblast shedding, which increases the amount of purine that is available for purine breakdown. Purine metabolite levels in the blood are being found higher in fetuses that have experienced hypoxia (for instance, as a result of diminished placental perfusion) (29). Therefore, the question of whether uric acid is only a disease marker or plays a causal role in the emergence of preeclampsia is still up for debate. Proteolytic processing of XDH during tissue ischemia takes place through cleavage of subunits and subsequent conversion to XO. During ischemic conditions, cells and cell organelles undergo necrosis which leads to the release of various enzymes from these organelles. It was found in a study (30) that this conversion is the consequence of leakage of mitochondrial proteins into the cytoplasm and ensuing XDH proteolysis. In another study (31) it was concluded that this conversion is also caused by modifications of cysteine residues by reagents such as nitrobenzoic acid or iodoacetamide. Since benzoic acid is being used as a food preservative, this could be a matter of debate for a risk factor for hyperuricemia. Some other factors could be the cause of hyperuricemia in pregnancy other than hyperactivity of XO which includes reduced renal clearance. Uric acid concentration is elevated as early as 10 weeks of gestation, a time much earlier than the clinical presentation of the PE. In a study (32) it was concluded that hypovolemia, an early change in preeclampsia, increases uric acid reabsorption which could increase serum uric acid concentrations.

Most eukaryotic cells contain lactate dehydrogenase (LDH), a cytosolic enzyme that is stable and soluble. LDH is released into the extracellular fluid upon necrosis (accidental cell death) or apoptosis when the plasma membrane is disrupted (programmed cell death). Cells that have been damaged lose the integrity of their membranes before shutting down and releasing their intracellular contents into the environment. To identify cellular damage in the present study, LDH levels were assessed in pregnant women with preeclampsia. The average LDH level in preeclampsia was higher than in control. Similar findings were made in a study by Dave et al. (33) who concluded that serum LDH is the first marker seen in the blood during hypoxia and oxidative stress. In the present study, uric acid and LDH levels were compared in both mild (48 females) and severe (52 females) cases and found that the correlation is positive (R = 0.2520, R = 0.2441), but didn't increase with severity. When correlation was drawn in overall 100 cases, it was more positive (R = 0.4066). These results suggest a low sample size. In the present study, it was found that uric acid had a high positive correlation with liver marker enzyme in mild preeclampsia as compared to severe preeclampsia. On other hand, LDH had a high positive correlation with ALT and AST in severe cases as compared to mild (Table 7; Figure 1). These findings indicate uric acid along with liver function tests can serve as a promising marker in early preeclampsia and LDH in later severe preeclampsia.

**Table 7:** Comparison of correlations in mild cases, severe cases, and total cases.

Correlation between	In mild preeclampsia	In severe preeclampsia	In total (Mild + Severe)
AST & ALT	0.7938	0.9107	0.8512
AST & UA	**0.5699**	0.2395	0.4618
AST& LDH	0.1897	**0.5424**	0.4242
ALT & UA	**0.5167**	0.3657	0.4917
ALT & LDH	0.2497	**0.5312**	0.4829
LDH & UA	0.2520	0.2441	0.4066
LDH & SBP	0.2010	0.1855	0.4438
UA & SBP	−0.1532	0.1577	0.3614

Showing a comparison of the R-value of Pearson's correlation between various parameters in 48 patients with mild preeclampsia, 52 patients with severe preeclampsia, and an overall total of 100 patients. Bold values indicate statistically strong correlation.

The study was innovative in that the serum uric acid level was correlated with the serum LDH levels to assess the cellular damage caused by oxidative stress. A positive correlation between serum uric acid and LDH level (R = 0.4066), which inferred that the overproduction of H_2_O_2_ from the purine metabolism step catalyzed by xanthine oxidase is somehow responsible for oxidative stress, which results in cellular damage in preeclampsia. Since oxidative stress can directly affect vascular tone and function, for instance by oxidizing proteins or nucleic acids. Nitric oxide (NO) non-bioavailability and/or signaling decline, which results in endothelial dysfunction, is a key mechanism for how oxidative stress affects vascular tone. It's possible that ROS will paradoxically produce the ROS superoxide anion rather than the reducing NO when eNOS catalyzes the reduction of molecular oxygen from the oxidation of L-arginine (33). As an alternative, ROS may directly interact with NO, lowering its bioavailability (22). Inflammation, apoptosis, and extracellular matrix modifications are further processes that ROS may support. One of the main complications of preeclampsia is hypertension, which can be caused by all of these ROS-related processes. Reports are available where increased apoptosis has been implicated in the pathogenesis of PE.

## 5 Conclusion

In this study, a significant positive association was found between serum uric acid, LDH, and systolic blood pressure. Therefore, we propose that serum uric acid throughout pregnancy be used to track the development of endothelial dysfunction brought on by oxidative stress, which can result in preeclampsia. Uric acid and liver function tests can be used for the early detection of preeclampsia. The sample size is just 100 females; thus, larger studies with more participants can be designed to provide additional light on the potential usage of Allopurinol during pregnancy to lower oxidative stress and to find any role of food preservatives (benzoic acid) in hyperuricemia. An experimental study (34) revealed the inhibitory effect of thiadiazole derivatives on LDH enzyme activity in thalassemia patients; therefore, an observational study should be conducted to find their effects in patients with preeclampsia.

## Data Availability

The datasets presented in this article are not readily available. Requests to access the datasets should be directed to simmikh@gmail.com.
